# A Double-Edged Sword Role for Ubiquitin-Proteasome System in Brain Stem Cardiovascular Regulation During Experimental Brain Death

**DOI:** 10.1371/journal.pone.0027404

**Published:** 2011-11-14

**Authors:** Carol H. Y. Wu, Julie Y. H. Chan, Samuel H. H. Chan, Alice Y. W. Chang

**Affiliations:** 1 Center for Translation Research in Biomedical Sciences, Kaohsiung Chang Gung Memorial Hospital, Taiwan, Republic of China; 2 Institute of Biomedical Sciences, National Sun Yat-sen University, Taiwan, Republic of China; University of South Florida College of Medicine, United States of America

## Abstract

**Background:**

Brain stem cardiovascular regulatory dysfunction during brain death is underpinned by an upregulation of nitric oxide synthase II (NOS II) in rostral ventrolateral medulla (RVLM), the origin of a life-and-death signal detected from blood pressure of comatose patients that disappears before brain death ensues. Furthermore, the ubiquitin-proteasome system (UPS) may be involved in the synthesis and degradation of NOS II. We assessed the hypothesis that the UPS participates in brain stem cardiovascular regulation during brain death by engaging in both synthesis and degradation of NOS II in RVLM.

**Methodology/Principal Findings:**

In a clinically relevant experimental model of brain death using Sprague-Dawley rats, pretreatment by microinjection into the bilateral RVLM of proteasome inhibitors (lactacystin or proteasome inhibitor II) antagonized the hypotension and reduction in the life-and-death signal elicited by intravenous administration of *Escherichia coli* lipopolysaccharide (LPS). On the other hand, pretreatment with an inhibitor of ubiquitin-recycling (ubiquitin aldehyde) or ubiquitin C-terminal hydrolase isozyme L1 (UCH-L1) potentiated the elicited hypotension and blunted the prevalence of the life-and-death signal. Real-time polymerase chain reaction, Western blot, electrophoresis mobility shift assay, chromatin immunoprecipitation and co-immunoprecipitation experiments further showed that the proteasome inhibitors antagonized the augmented nuclear presence of NF-κB or binding between NF-κB and *nos II* promoter and blunted the reduced cytosolic presence of phosphorylated IκB. The already impeded NOS II protein expression by proteasome inhibitor II was further reduced after gene-knockdown of NF-κB in RVLM. In animals pretreated with UCH-L1 inhibitor and died before significant increase in *nos II* mRNA occurred, NOS II protein expression in RVLM was considerably elevated.

**Conclusions/Significance:**

We conclude that UPS participates in the defunct and maintained brain stem cardiovascular regulation during experimental brain death by engaging in both synthesis and degradation of NOS II at RVLM. Our results provide information on new therapeutic initiatives against this fatal eventuality.

## Introduction

Brain death, the legal definition of death in many countries [1]–[3], is commonly recognized as a neurological phenomenon. Two clinical observations, however, in effect place brain death into the realm of circulatory research. First, asystole invariably takes place within hours or days after the diagnosis of brain death [4]. Second, a unique prognostic phenotype for life-and-death exists in the low-frequency (LF) component (0.04–0.15 Hz in human) of systemic arterial pressure (SAP) spectrum. The power density of the LF component, which mirrors the prevalence of baroreflex-mediated sympathetic neurogenic vasomotor tone [5], undergoes a dramatic reduction or loss before brain death ensues in comatose patients [6]–[8] to reflect irreversible failure of brain stem cardiovascular regulatory functions [9]. It follows that delineation of the mechanisms that underpin the shift between maintained and defunct brain stem cardiovascular regulatory machinery during the progression towards brain death should shed further light on this fatal phenomenon. That the LF component originates from the rostral ventrolateral medulla (RVLM) [10], which is known classically for its role in tonic maintenance of vasomotor tone and SAP [11], allows this brain stem site to be a suitable neural substrate for such a mechanistic delineation [9].

It is now clear that most proteins in the cytoplasm and nucleus of eukaryotic cells are degraded via the ubiquitin-proteasome system (UPS) [12], [13]. The highly conserved 76 amino acid protein ubiquitin is best known for its role in targeting proteins for degradation by the 26S proteasome. Conjugation of ubiquitin to the protein substrate during ubiquitination proceeds via a three-step mechanism. The ubiquitin-activating enzyme, E1, first activates ubiquitin. Following activation, one of several ubiquitin-conjugating enzymes (E2) transfers ubiquitin from E1 to a member of the ubiquitin-protein ligase family (E3), to which the substrate protein is specifically bound. Polyubiquitinated proteins are recognized by the regulatory 19S complexes of the proteasome, which unfold the protein substrates and assist in their translocation through a narrow gate into the 20S core where degradation takes place. Following conjugation, the protein moiety of the adduct is degraded by the 26S proteasome complex. After the degradative process at the 26S proteasome, the ubiquitin chain is released from the target protein remnant and is disassembled by de-ubiquitinating enzymes, including the ubiquitin C-terminal hydrolases (UCHs) [14]. The UCHs are responsible for the removal of small peptide fragments from the ubiquitin chain and for co-translational processing of ubiquitin gene products to generate free monomeric ubiquitin [14], [15]. Of the three known mammalian members of the UCH family, UCH isozyme-L1 (UCH-L1) is among the most abundantly present proteins in brain [16]. One of the best-known targets of the UPS is activation of the inducible transcription factor nuclear factor-κB (NF-κB) [17], [18]. NF-κB is retained in a latent form in the cytoplasm of non-stimulated cells by inhibitory molecules collectively termed inhibitory-κB (IκB). Stimuli that induce NF-κB activation target IκB to site-specific phosphorylation, leading to its degradation by the UPS. Following IκB degradation, NF-κB is translocated to the nucleus as an active transcription factor that is able to induce its target genes.

In an animal model that employs *Escherichia coli* lipopolysaccharide (LPS) as the experimental insult [19]–[21], our laboratory found previously that the dysfunction of brain stem cardiovascular regulatory machinery as reflected by the reduction in the power density of the LF component during the advancement towards brain death is associated with the progressive augmentation in both molecular synthesis and functional expression of nitric oxide synthase II (NOS II) in RVLM. We further showed that transcriptional regulation by NF-κB is crucial to the expression of NOS II gene [20]. At the same time, scattered reports suggest that the UPS may also be involved in the degradation of NOS II [22], [23]. It follows that the UPS may participate actively in brain stem cardiovascular control during brain death by engaging in both the synthesis and degradation of NOS II in RVLM. This hypothesis is validated based on an experimental endotoxemia model.

## Results

### UPS in RVLM participates in brain stem cardiovascular regulation during experimental brain death

**Figure 1:**
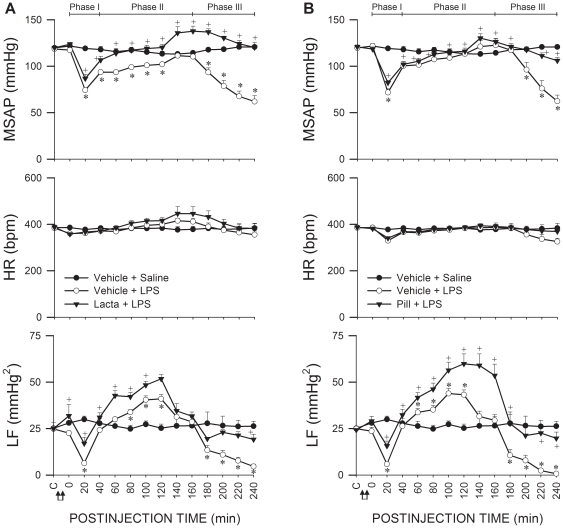
UPS in RVLM participates in brain stem cardiovascular regulation during experimental brain death. Temporal changes in mean systemic arterial pressure (MSAP), heart rate (HR) or power density of the low-frequency (LF) component of SAP signals in rats that received pretreatment by microinjection bilaterally into RVLM of lactacystin (Lacta; 1 nmol) (A), proteasome inhibitor II (PiII; 1 nmol) (B) or their solvents (Vehicle), followed by IV administration (at arrows) of LPS (15 mg kg^−1^) or saline. Values are mean ± SEM; n = 7–8 animals per group. *P <0.05 versus Vehicle+Saline group, and ^+^ *P* <0.05 versus Vehicle+LPS group at corresponding time-points in the Scheffé multiple-range test. C, baseline. Demarcation of Phases I, II and III according to the decrease, increase and secondary decrease of LF power is shown on top of the diagrams.

An experimental endotoxemia model of brain death [9] that mimics clinically the progression towards brain death in patients died of systemic inflammatory response syndrome [7] was used. As reported previously [19]–[21], based on the decrease, increase, and a secondary decrease in the power density of the LF component in the SAP spectrum, which reflect lower or higher probability of defunct or maintained brain stem circulatory regulation, the sequence of cardiovascular responses induced by intravenous administration of LPS (15 mg kg^−1^) can be divided into three phases (Figure 1). SAP underwent typically a significant decrease and a rebound during Phase I, to be followed by progressive hypotension during Phases II and III endotoxemia. Fluctuations in heart rate (HR) were insignificant. Intriguingly, inhibiting proteasome activities by microinjection bilaterally of a non-selective proteasome inhibitor [24], lactacystin (1 nmol; Figure 1A) or a specific inhibitor of chymotrypsin-like proteasomal activity [25], proteasome inhibitor II (1 nmol; Figure 1B) into RVLM significantly antagonized the elicited hypotension. The increase in power density of LF component of SAP signals during Phase II endotoxemia was potentiated, and the decrease in LF power during Phase III was significantly blunted.

### Drastic difference between upregulation of NOS II mRNA and protein in RVLM during experimental brain death

**Figure 2:**
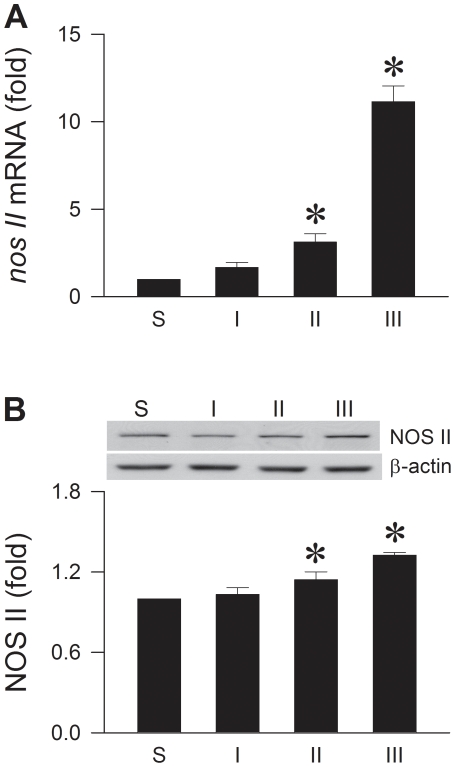
Drastic difference between upregulation of NOS II mRNA and protein in RVLM. Illustrative gels or summary of fold changes against sham-controls in *nos II* mRNA determined by real-time PCR (A) or NOS II protein detected by Western blot analysis (B) in ventrolateral medulla of rats that received IV administration of LPS (15 mg kg^−1^). Values are mean ± SEM of triplicate (mRNA) or duplicate (protein) analyses on individual samples obtained from 5–6 animals per experimental group. *P <0.05 versus sham-control group in the Scheffé multiple-range test.

Our laboratory demonstrated previously [19]–[21] that upregulation of NOS II in RVLM underlies brain stem cardiovascular regulatory dysfunction in our experimental endotoxemia model of brain death. Whether this NOS isoform is a target for post-translational modification by the UPS was investigated in our second series of experiments. Real-time polymerase chain reaction (PCR) analysis revealed significant and progressive augmentation of *nos II* mRNA in RVLM, reaching approximately 22 folds over sham-controls during Phase III (Figure 2A). Whereas protein expression of NOS II as determined by Western-blot analysis also underwent a gradual increase (Figure 2B), it amounted to only 1.3 folds over sham-controls during Phase III.

### UPS is engaged in the synthesis of NOS II at RVLM

**Figure 3:**
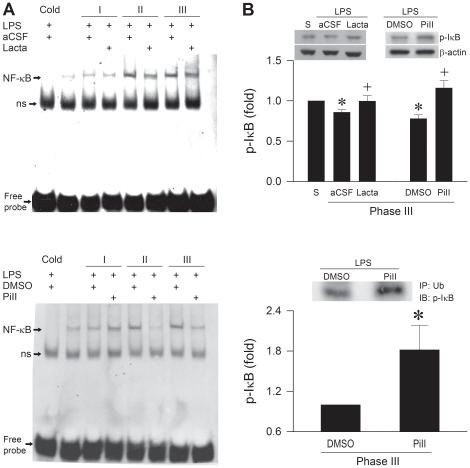
UPS is engaged in the synthesis of NOS II via transcriptional activation of NF-κB in RVLM. (A) Representative gels depicting NF-κB DNA binding detected by EMSA in nuclear extracts from ventrolateral medulla of rats that received pretreatment by microinjection bilaterally into RVLM of lactacystin (Lacta; 1 nmol) or aCSF (upper diagram), or proteasome inhibitor II (PiII; 1 nmol) or DMSO (lower diagram), followed by IV administration of LPS (15 mg kg^−1^). A competitive assay with the addition of 100-fold of unlabeled NF-κB oligonucleotide was used to control for non-specific binding (Cold). In both gels, ns denotes non-specific binding. (B) Illustrative gels or summary of fold changes against sham-controls of phosphorylated IκBα (p-IκBα) in cytosolic proteins detected by Western blot analysis (upper diagram) or immunoblot analysis of p-IκBα immunoprecipitated by an anti-ubiquitin (Ub) antiserum (lower diagram) during Phase III experimental endotoxemia in rats that received those treatments depicted in (A). Values are mean ± SEM of duplicate analyses on individual samples obtained from 5–6 animals per experimental group. *P <0.05 versus sham-control group, and ^+^ *P* <0.05 versus aCSF+LPS or DMSO+LPS group in the Scheffé multiple-range test.

**Figure 4:**
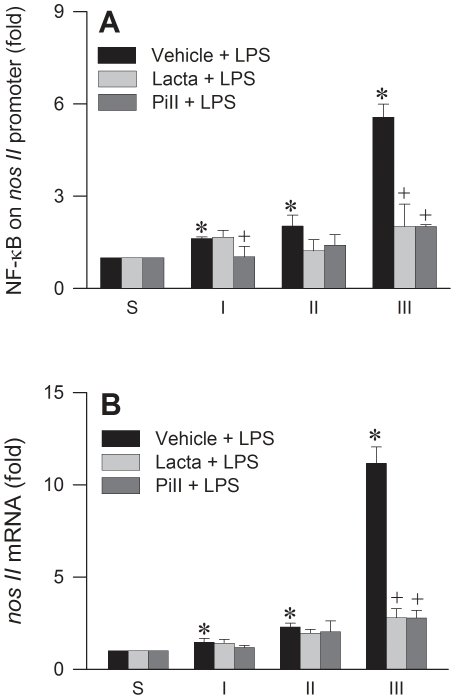
NF-κB transcriptionally activated by UPS binds to *nos II* promoter in RVLM. Phasic fold changes against sham-controls in (A) binding between NF-κB and *nos II* promoter measured by ChIP assay, or (B) *nos II* mRNA level determined by real-time PCR analysis in ventrolateral medulla of rats that received pretreatment by microinjection bilaterally into RVLM of lactacystin (Lacta; 1 nmol), proteasome inhibitor II (PiII; 1 nmol) or their solvent (vehicle), followed by IV administration of LPS (15 mg kg^−1^). Values are mean ± SEM of triplicate analyses on individual samples obtained from 5–6 animals per experimental group. *P <0.05 versus sham-control group, and ^+^P <0.05 versus Vehicle+LPS group at corresponding phases in the Scheffé multiple-range test.

An upregulation of NOS II through NF-κB activation in RVLM [20] plays a pivotal role in our experimental endotoxemia model of brain death. As such, the UPS may participate in the synthesis of NOS II via its regulatory role in NF-κB activation [17], [18]. Results from electrophoresis mobility shift assay (EMSA) (Figure 3A) showed a significant and progressive increase in the association of NF-κB with its consensus DNA oligonucleotide in nuclear extracts from RVLM during experimental brain death. We confirmed that this association was not due to non-specific binding when competitive assay using unlabeled NF-κB oligonucleotide resulted in appreciable disappearance of NF-κB DNA binding (Figures 3A and S1). Supershift experiments further revealed specific binding by showing that an anti-NFκB p50 antiserum retarded the migration of proteins that interacted with the NF-κB oligonucleotide (Figure S1). Intriguingly, microinjection bilaterally of lactacystin (1 nmol) or proteasome inhibitor II (1 nmol) into RVLM discernibly antagonized the augmented nuclear presence of NF-κB (Figure 3A). In addition, both proteasome inhibitors significantly antagonized the reduced phosphorylated IκB (pIκB) level in cytosolic fraction of extracts from RVLM during Phase III (Figure 3B). Co-immunoprecipitation experiments also indicated that the association between ubiquitin and pIκB in the cytosol was significantly strengthened in the presence of proteasome inhibitor II (Figure 3B). Results from chromatin immunoprecipitation (ChIP) assay using an anti-NFκB p65 antiserum further ascertained a progressively augmented binding between NF-κB and *nos II* promoter in RVLM, which was significantly antagonized by lactacystin or proteasome inhibitor II (Figure 4A). This implicated crucial involvement of UPS-mediated IκB degradation in the synthesis of NOS II was further established by comparable results at *nos II* mRNA level based on real-time PCR analysis (Figure 4B).

### UPS is also engaged in the degradation of NOS II at RVLM

**Figure 5:**
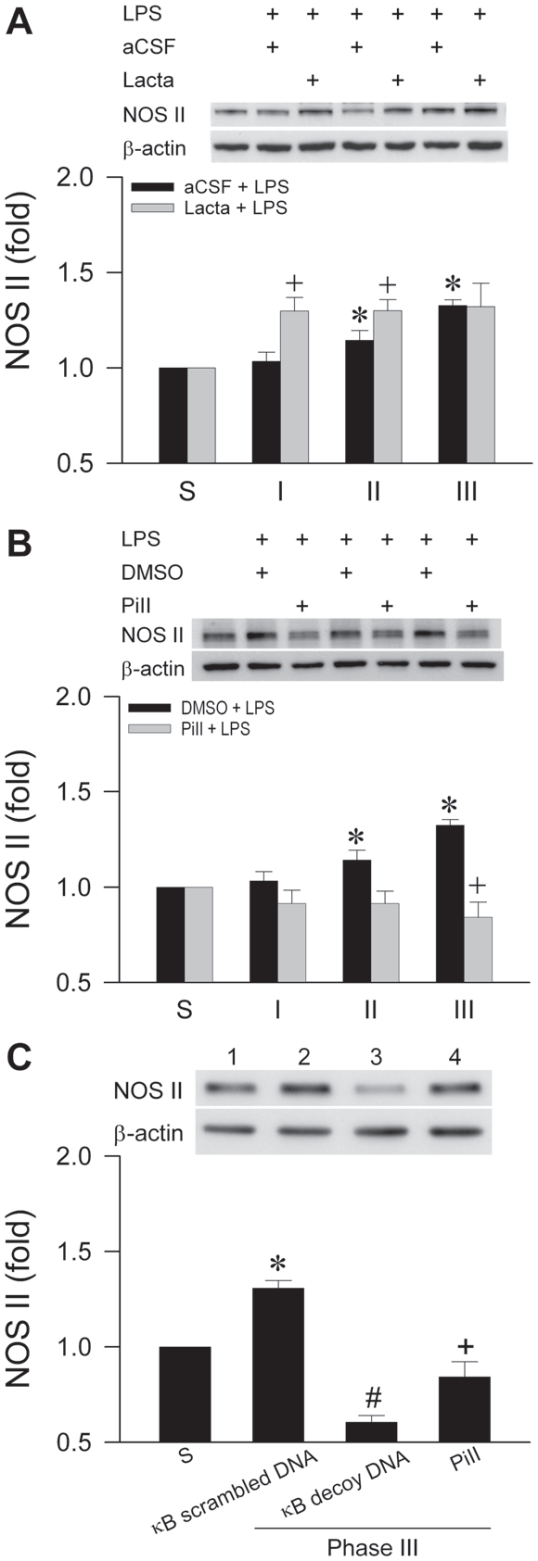
UPS is also engaged in the degradation of NOS II at RVLM. Illustrative gels or summary of fold changes against sham-controls of NOS II proteins detected by Western blot analysis in ventrolateral medulla of rats that received pretreatment by microinjection bilaterally into RVLM of lactacystin (Lacta; 1 nmol) or aCSF (A), proteasome inhibitor II (PiII; 1 nmol) or DMSO (B) or scrambled or decoy κB DNA (C), followed by IV administration of LPS (15 mg kg^−1^). Values are mean ± SEM of duplicate analyses on individual samples obtained from 5–6 animals per experimental group. *P <0.05 versus sham-control group, and ^+^ *P* <0.05 versus aCSF+LPS or DMSO+LPS group at corresponding phases (A, B); or *P <0.05 versus sham-control group, ^#^ *P* <0.05 versus PiII+LPS group and ^+^ *P* <0.05 versus scrambled κB DNA +LPS group (C) in the Scheffé multiple-range test.

Our fourth series of experiments delineated whether the UPS is also engaged in the degradation of NOS II in RVLM, as suggested by the drastic disparity of the upregulated mRNA and protein levels. Figure 5A shows that in the presence of lactacystin (1 nmol), the protein expression of NOS II in RVLM during Phases I and II was significantly augmented, although there was no further increase during Phase III experimental brain death. Pretreatment with proteasome inhibitor II (1 nmol) completely antagonized the progressive increase in NOS II protein expression (Figure 5B). Intriguingly, gene-knockdown of NF-κB with κB decoy DNA not only blunted the significantly augmented NOS II protein expression when compared to κB scrambled DNA controls, but further reduced it to a level that was significantly below that in the proteasome inhibitor II-pretreatment group (Figure 5C).

### De-ubiquitination is crucially involved in maintaining brain stem cardiovascular regulation during experimental brain death

**Figure 6:**
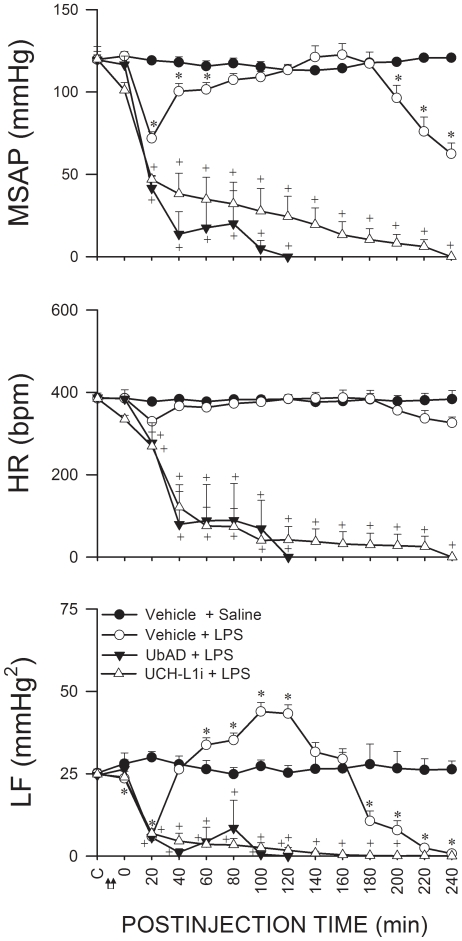
De-ubiquitination is crucially involved in maintaining brain stem cardiovascular regulation during experimental brain death. Temporal changes in MSAP, HR or power density of the LF component of SAP signals in rats that received pretreatment by microinjection bilaterally into RVLM of ubiquitin aldehyde (UbAd), UCH-L1 inhibitor (UCH-L1i) or their solvents (Vehicle), followed by IV administration (at arrows) of LPS (15 mg kg^−1^) or saline. Values are mean ± SEM; n = 7–8 animals per group. *P <0.05 versus Vehicle+Saline group, and ^+^ *P* <0.05 versus Vehicle+LPS group at corresponding time-points in the Scheffé multiple-range test. C, baseline.

Pretreatment by microinjection bilaterally into RVLM of a general inhibitor of ubiquitin-recycling [26], ubiquitin aldehyde (50 fmol) or a potent, reversible, competitive and active site-directed UCH-L1 inhibitor [27], (1 nmol), significantly potentiated the elicited hypotension, initiated bradycardia and blunted the increase in power density of LF component during the significantly shortened Phase II experimental brain death (Figure 6).

### De-ubiquitination is crucially involved in degradation of NOS II at RVLM

**Figure 7:**
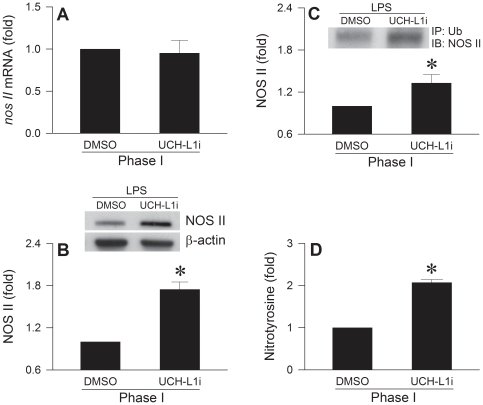
De-ubiquitination is crucially involved in degradation of NOS II at RVLM. Illustrative gels or summary of fold changes against sham-controls in *nos II* mRNA determined by real-time PCR (A), NOS II protein detected by Western blot analysis (B), immunoblot analysis of NOS II immunoprecipitated by an anti-ubiquitin (Ub) antiserum (C) or nitotyrosine determined by ELISA (D) in ventrolateral medulla of rats that received pretreatment by microinjection bilaterally into RVLM of UCH-L1 inhibitor (UCH-L1i) or DMSO, during Phase I experimental endotoxemia induced by IV administration (at arrows) of LPS (15 mg kg^−1^). Values are mean ± SEM of triplicate (mRNA) or duplicate (protein) analyses on individual samples obtained from 5–6 animals per experimental group. *P <0.05 versus DMSO-control group in the Scheffé multiple-range test.

Our final series of experiments determined whether de-ubiquitination is more engaged in degradation or synthesis of NOS II. Animals that received pretreatment with UCH-LI inhibitor (1 nmol) and succumbed to LPS within 5–10 min after administration demonstrated that in the absence of significant increase in *nos II* mRNA (Figure 7A); NOS II protein level in RVLM was augmented by more than 50% (Figure 7B). There was also significantly elevated ubiquitinylated NOS II in RVLM (Figure 7C), alongside augmented nitrotyrosine (Figure 7D), an experimental index for peroxynitrite that underlies fatality in experimental brain death [20], [21].

## Discussion

**Figure 8:**
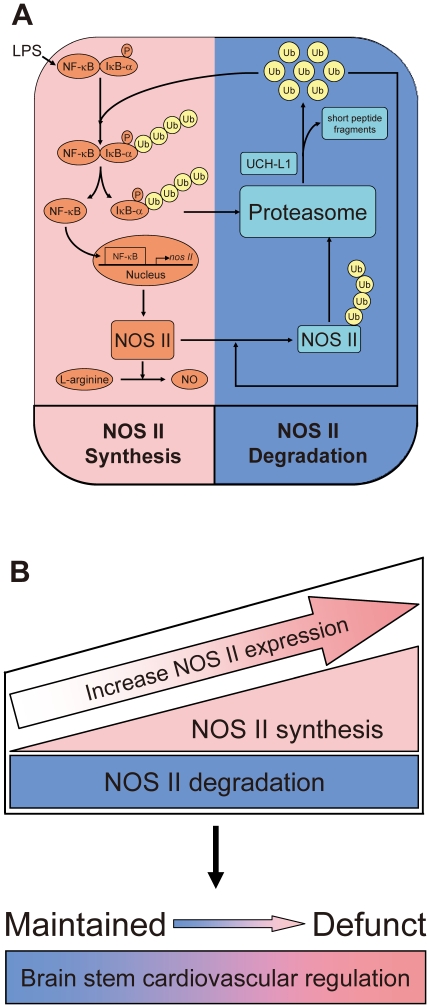
Schematic illustration of a double-edge sword role for the UPS in brain stem cardiovascular regulation during experimental brain death. A crucial determinant in the tug-of-war between maintained and defunct brain stem circulatory control resides in the balance between degradation and synthesis (via NF-κB activation) of NOS II, both processes of which require augmented ubiquitination, enhanced proteasome activities and de-ubiquitination in RVLM.

The fundamental premise of the present study is that the progression from maintained to defunct brain stem cardiovascular regulation during the advancement of brain death is underpinned by the progressive increase in NOS II level in RVLM [19]–[21]. Based on an endotoxemia model of experimental brain death, our results revealed a novel, causal and double-edged sword role for the UPS at RVLM in this process (Figure 8A). Specifically, we demonstrated that a crucial determinant in the tug-of-war between maintained and defunct brain stem circulatory regulation during brain death resides in the temporal balance between the continuous degradation of NOS II and the progressively augmented synthesis of the isozyme in RVLM (Figure 8B). We further found that recycling of ubiquitin in RVLM through sustained de-ubiquitination is crucial to uninterrupted degradation of NOS II, which is essential for the maintenance of brain stem cardiovascular control.

Our laboratory demonstrated previously that an upregulation of NOS II through NF-κB activation at RVLM [20] plays a pivotal role in eliciting defunct brain stem cardiovascular regulation during experimental endotoxemia. The *nos II* gene is potentially regulated transcriptionally by NF-κB [28]–[30] because NF-κB-binding sites are present in the rat [29] or mouse [31] *nos II* promoter, and are considered to be critical for gene expression. Of note is that activation of NF-κB is one of the best-known targets of the UPS [17], [18]. Site-specific phosphorylation of IκB, which leads to its degradation by the UPS, allows NF-κB to be translocated to the nucleus as an active transcription factor that is able to induce its target genes. Our results from EMSA, ChIP assay, co-immunoprecipitation and real-time PCR showed that this repertoire of cellular events indeed takes place during experimental brain death, leading to the synthesis of NOS II in RVLM.

The physiological importance of degradation of NOS II by the UPS has been shown in Dahl/Rapp salt-sensitive rats [23]. The 26S proteasome was identified [32] to be the primary degradation site for NOS II in human epithelial kidney HEK293 cells and murine macrophage cell line RAW 264.7; ubiquitination is required for this degradative process [22]. It is therefore conceivable that continuous degradation by the UPS may account for the moderate increase in NOS II protein despite drastically augmented *nos II* mRNA in RVLM during experimental brain death. Whereas our observations after lactacystin pretreatment (Figure 5A) support this notion, our results from proteasome inhibitor II (Figure 5B) suggest otherwise. Furthermore, pretreatments with proteasome inhibitors may prevent NOS II synthesis in RVLM by reducing IκB degradation, leading to an increase in association between NF-κB and IκB in the cytoplasm, which prevents nuclear translocation of NF-κB. This riddle was resolved by knockdown of NF-κB gene with κB decoy DNA (Figure 5C). In the absence of NOS II synthesis via NF-κB activation, the significant reduction from the already impeded NOS II protein expression in RVLM of the proteasome inhibitor II-pretreatment group lends support to the notion that degradation of NOS II indeed takes place in RVLM during experimental brain death. The differential effects of lactacystin and proteasome inhibitor II also infer that chymotrypsin-like activity in the proteasome may be primarily responsible for the degradation of NOS II in RVLM.

Our laboratory found previously that peroxynitrite formed by a reaction between NOS II-derived NO and superoxide anion underlies the dysfunctional brain stem cardiovascular regulation in our experimental endotoxemia model of brain death [20], [21]. Intriguingly, we found that whereas degradation of NOS II protein was maintained throughout our 240-min observation period, synthesis of this isoform via NF-κB activation following IκB degradation became prevalent only during Phases II and III. The resultant progressive increase in NOS II protein in RVLM, which favors the production of peroxynitrite that leads to cardiovascular depression, is commensurate with the observation that pretreatment with the proteasome inhibitors significantly antagonized the progressive hypotension and decrease in LF power, and improved survival rate during experimental brain death. We are aware that overexpression of NOS II in RVLM reportedly induces pressor and sympathoexcitatory response [33]. As such, the possibility exists for the initial increase in NOS II expression in RVLM to underpin the elevated LF power during Phase II endotoxemia.

Optimal operation of polyubiquitination also depends on the availability of monoubiquitin (free form of ubiquitin), which is regulated by the de-ubiquitinating enzymes [14]. By showing the effectiveness of ubiquitin aldehyde at fmol concentration to exacerbate cardiovascular depression, the present study revealed that recycling of ubiquitin in RVLM is vitally involved in the maintenance of brain stem cardiovascular regulatory functions in our experimental endotoxemia model of brain death. Of the known de-ubiquitination enzymes, UCH-L1 is among the most abundantly present proteins in brain [16]. The present study showed that on an equimolar basis (1 nmol), the degree of amelioration of defunct brain stem cardiovascular regulation by lactacystin or proteasome inhibitor II was significantly less than the detrimental effects of UCH-L1 inhibitor. This result is interpreted to imply that de-ubiquitination by UCH-L1 in RVLM plays a maintenance role in brain stem cardiovascular regulation during experimental brain death. Animals in which recycling of ubiquitin in RVLM was inhibited and died within 5–10 min of LPS administration provided a golden opportunity to delineate the underlying mechanism. The 50% increase in NOS II protein expression in RVLM at a time when there was no significant increase in *nos II* mRNA, alongside elevated ubiquitinylated NOS II and augmented nitrotyrosine, amply supports the notion that the free forms of ubiquitin availed by UCH-L1 is targeted for degradation of NOS II. In addition, UCH-L1 may also reduce NOS II synthesis by attenuation of NF-κB activation [34].

We are cognizant that LPS also activates NF-κB that leads to the synthesis of NOS II in peripheral tissues [30]. In this regard, our clinical experience [7] is that a dramatic reduction or loss of the power density of the LF component consistently takes place before significant hypotension occurs during the progression towards brain death in patients who succumbed to systemic inflammatory response syndrome, followed by asystole. This suggests that synthesis of NOS II via activation of NF-κB in RVLM, leading to a reduction in LF power that results in defunct brain stem cardiovascular regulation may play a pivotal role in our experimental endotoxemia model of brain death. The design of our study did not allow us to decipher the cell types in RVLM that were affected by LPS. Nevertheless, previous studies from our laboratory [35] showed that NOS II is activated by LPS in neurons, astrocytes and microglia in RVLM.

The UPS has emerged in recent years as a central player in the modulation of cell fate [36] and cell death pathways [37]. Aberrations in either the process of ubiquitination or de-ubiquitination have also been directly implicated in the etiology of many diseases [13], including cardiomyopathy [38]. It is therefore of interest that the present study provided novel findings to support the notion that the UPS participates in the defunct and maintained brain stem cardiovascular regulation by engaging in both synthesis and degradation of NOS II at RVLM during experimental brain death. We further found that recycling of ubiquitin in RVLM through sustained de-ubiquitination is vitally important. This information shall be invaluable to future development of management strategies against fatal eventualities such as brain death. It also posts a cautionary note on using the UPS as a therapeutic target because of the potential double-edged sword actions on its protein substrates.

## Materials and Methods

### Ethics Statement

All experimental procedures carried out in this study have been approved by the Institutional Animal Care and Use Committees of the National Sun Yat-sen University and Kaohsiung Chang Gung Memorial Hospital (#96008), and were in compliance with the guidelines for animal care and use set forth by those committees. All efforts were made to minimize animal suffering and to reduce the number of animal used.

### Animals

Adult, male Sprague-Dawley rats (202−255 g; n = 518) were purchased from the Experimental Animal Center of the National Applied Research Laboratories, Taiwan. They were housed in an Association for Assessment and Accreditation of Laboratory Animal Care (AAALAC) International-accredited animal facility under temperature control (24 ± 0.5°C) and 12-h light-dark cycle (lights on during 08:00–20:00). Standard laboratory rat chow and tap water were available ad libitum. Animals were allowed to acclimatize for at least 7 days prior to experimental manipulations.

### General preparation

Under initial pentobarbital sodium anesthesia (50 mg kg^−1^, IP), the trachea was intubated and the right femoral artery and both femoral veins were cannulated. Animals received thereafter IV infusion of propofol (Zeneca, Macclesfield, UK) at 20 mg kg^−1^ h^−1^. This scheme provides satisfactory anesthetic maintenance while preserving the capacity of brain stem cardiovascular regulation [39]. The head of animals was fixed to a stereotaxic headholder (Kopf, Tujunga, CA), and body temperature was maintained at 37°C with a heating pad. Animals were allowed to breathe spontaneously with room air during the recording session.

### Recording and power spectral analysis of systemic arterial pressure signals

SAP signals recorded from the femoral artery were subject to on-line and real-time power spectral analysis [40]. HR was estimated instantaneously from the digitized SAP signals. We were particularly interested in the LF component (0.25–0.8 Hz) in the SAP spectrum for three reasons. First, its power density mirrors the prevalence of baroreflex-mediated sympathetic neurogenic vasomotor discharges [5] that emanate from this brain stem site [11] and is therefore a reasonable index for brain stem cardiovascular regulation. Second, it takes origin from RVLM [10]. Third and most importantly, the power density of the LF component represents the most crucial link between our animal model and clinical observations from patients who died of systemic inflammatory response syndrome [7], and is a more sensitive prognostic index than SAP for brain death [9].

### Experimental endotoxemia model of brain death

An experimental endotoxemia model of brain death [9], which mimics clinically the progression towards brain death in patients died of systemic inflammatory response syndrome [7] was used. *Escherichia coli* LPS (serotype 0111:B4, Sigma-Aldrich, St. Louis, MO) was administered intravenously (15 mġkg^−1^), with saline serving as the vehicle control. Temporal changes in pulsatile SAP, mean SAP (MSAP), HR and power density of the LF component were routinely followed for 240 min, in an on-line and real-time manner [40]. As we reported previously [20], [21], [41], the sequence of cardiovascular events during this LPS-induced endotoxemia can be divided into a reduction (Phase I), followed by an augmentation (Phase II) and a secondary decrease (Phase III) in the power density of the LF component of SAP signals. This triphasic change in LF power can be used to reflect lower or higher probability of defunct or maintained brain stem circulatory regulation during experimental brain death. There was also progressive hypotension, with death ensuing generally within 4 h in approximately 40% of the animals.

### Microinjection of test agents into RVLM

To produce site-specific actions, test agents were microinjected bilaterally and sequentially into RVLM via a glass micropipette connected to a 0.5-μl Hamilton (Reno, NV) microsyringe [5], [20], [21], [41]. The coordinates used were: 4.5 to 5 mm posterior to the lambda, 1.8 to 2.1 mm lateral to the midline and 8.1 to 8.4 mm below the dorsal surface of the cerebellum. These coordinates were selected to cover the ventrolateral medulla at which functionally identified sympathetic premotor neurons reside. Functional location of RVLM neurons was carried out at the beginning of each experiment by the elicitation of a transient increase in SAP (15–20 mmHg) on microinjection of glutamate. As a routine, a total volume of 50 nl was delivered to each side of RVLM over 2–3 min to allow for complete diffusion of the test agents.

Test agents used included a non-selective proteasome inhibitor [24], lactacystin (Calbiochem, San Diego, CA); a specific inhibitor of chymotrypsin-like proteasomal activity [25], proteasome inhibitor II (Calbiochem); a general inhibitor of ubiquitin-recycling [26], ubiquitin aldehyde (Calbiochem); a potent, reversible, competitive and active site-directed inhibitor of UCH-L1 [27], (Calbiochem); and double-stranded κB decoy DNA (5’-GAGGGGACTTTCCCT-3’) or its scrambled sequence (5’-GATGCGTCTGTCGCA-3’) (Quality Systems, Taipei, Taiwan) [20]. The doses were adopted from previous reports that used those test agents for the same purpose as in this study. Test agents were dissolved in artificial cerebrospinal fluid (aCSF), with the exception of proteasome inhibitor II and UCH-L1 inhibitor, which was dissolved in 10% and 40% DMSO. Possible volume effect of microinjection was controlled by injecting the same amount of solvent. All test agents or their vehicles were given 30 min before LPS administration, with the exception that double-stranded κB decoy DNA or its scrambled sequence was given 24 h prior to LPS treatment. To avoid the confounding effects of drug interactions, each animal received only one test agent.

### Collection of tissue samples from RVLM

We routinely collected tissue samples from RVLM [20], [21], [41] at the peak of each phase of experimental endotoxemia (LPS group) or 15 min, 1.5 h or 3 h after IV injection of saline (vehicle group); or immediately after animals succumbed to our experimental treatments. Medullary tissues collected from anesthetized animals but without treatment served as sham-controls. As a routine, microinjection sites were visually verified and recorded after the slice of medulla oblongata that contains RVLM (0.5 to 1.5 mm rostral to the obex) was obtained. Tissues from both sides of the ventrolateral medulla were subsequently collected by micropunches made with a 1 mm (id) stainless steel bore to cover the anatomical boundaries of RVLM. In some experiments, proteins from the nuclear or cytosolic fraction of the medullary samples were extracted by a commercial kit (Active Motif, Carlsbad, CA). The concentration of total or fractional proteins extracted was determined by the BCA Protein Assay (Pierce, Rockford, IL).

### Western blot analysis

Western blot analysis [20], [21], [41]–[44] was carried out using a rabbit polyclonal antiserum against NOS II (Santa Cruz, Santa Cruz, CA); or a mouse monoclonal antiserum against p-IκBα (Santa Cruz) or β-actin (Chemicon, Temecula, CA). This was followed by incubation with horseradish peroxidase-conjugated donkey anti-rabbit IgG (Amersham Biosciences, Little Chalfont, Bucks, UK) for NOS II; or sheep anti-mouse IgG (Amersham Biosciences) for p-IκBα or β-actin. Specific antibody-antigen complex was detected by an enhanced chemiluminescence Western blot detection system (Santa Cruz). The amount of protein was quantified by the ImageMaster Video Documentation System software (Amersham Biosciences, City, NJ), and was expressed as the ratio relative to β-actin protein.

### Immunoprecipitation and immunoblot analysis

Protein extracts from the cytosolic fraction of samples from RVLM were immunoprecipitated with affinity-purified mouse monoclonal anti-ubiquitin antiserum conjugated with protein G-agarose beads (Roche Diagnostics, Mannheim, Germany). Immunoprecipitation was performed [42] at 4°C overnight and the precipitated beads obtained after being centrifuged for 5 s at 6,000 *g* were washed three times with ice-cold lysis buffer. The agarose beads resuspended in the loading buffer were boiled for 5 min to dissociate the immunocomplexes from the beads. Western blot analysis of p-IκB or NOS II from proteins immunoprecipitated by an anti-ubiquitin antiserum was carried out as described above.

### Isolation of RNA and real-time PCR

Total RNA from RVLM was isolated with TRIzol reagent (Invitrogen, Carlsgad, CA) [20], [21], [41]–[43]. All RNA isolated was quantified by spectrophotometry and the optical density (OD) 260/280 nm ratio was determined. As in our previous studies [41]–[43], reverse transcriptase reaction was performed using a SuperScript Preamplification System (Invitrogen) for the first-strand cDNA synthesis. Real-time PCR analysis was performed by amplification of cDNA using a LightCycler^®^ instrument (Roche). PCR reaction for each sample was carried out in duplicate for all the cDNA and for the GAPDH control. Primers were designed using the sequence information of the NCBI database by Roche LightCycler^®^ probe design software 2.0, and oligonucleotides were synthesized by Genemed Biotechnologies (Taipei, Taiwan).

The primer pairs used for amplification of target genes were:

*nos II*: 5’-TGGAGGTGCTGGAAGAGTT-3’ (forward primer) and 5’-GGAGGAGCTGATGGAGTAGT-3’ (reverse primer);

GAPDH: 5’-GCCAAAAGGGTCATCATCTC-3’ (forward primer) and 5’-GGCCATCCACAGTCTTCT-3’ (reverse primer).

Fluorescence signals from the amplified products were quantitatively assessed using the LightCycler^®^ software program (version 3.5; Roche). Second derivative maximum mode was chosen with baseline adjustment set in the arithmetic mode. The relative changes in *nos II* mRNA expression were determined by the fold-change analysis [41], [44], in which Fold change  = 2^−[^δδ^Ct]^, where δδCt  = (Ct_nos II_ − Ct_GAPDH_)_LPS treatment_ − (Ct_nos II_ − Ct_GAPDH_)_sham control_). Note that Ct value is the cycle number at which fluorescence signal crosses the threshold.

### Nuclear protein extraction and EMSA

We employed EMSA [20], [44] to measure NF-κB DNA binding activity in nuclear proteins pooled from ventrolateral medulla of 5 to 6 rats. The 3’ end of a double-stranded synthetic oligonucleotide probe for NF-κB (5’-AGTGAGGGGACTTTCCCAGGC-3’ and 3’-TCAACTCCCCTGAAAGGGTCCG-5’) [20], [44] was labeled with digoxigenin-11-ddUTP (Roche). DNA and protein complexes resolved on 4% polyacrylamide gels by electrophoresis were detected by chemiluminescence after reacting with an anti-digoxigenin antiserum (Roche). Competitive assay using unlabeled NF-κB oligonucleotide served as the negative control. Antiserum against NF-κB p50 subunit was used in supershift assay to confirm specificity of NF-κB translocation to the nucleus.

### ChIP assay

ChIP assay was carried out using the EpiQuik™ tissue chromatin immunoprecipitation kit (Epigentek Group, Brooklyn, NY) according to the protocol recommended by the manufacturer and modified for brain tissues. RVLM slices were cross-linked in 1% formaldehyde at room temperature for 15–20 min on a rocking platform, followed by incubation with 0.125 M glycine for 5 min to stop the fixation. After washing using ice-cold PBS and homogenizing the tissues, cell lysates were sonicated on ice until the cross-linked chromatins are sheared to yield DNA fragments between 200 and 1000 base pairs. The supernatant (sonicated DNA) was diluted to the required volume with ChIP dilution buffer at a 1:1 ratio, and 5 µl of the diluted supernatant was removed to a 0.5 ml vial as “input” DNA and put on ice. Sonicated DNA (500 µg) taken from each sample was incubated with an anti-NFκB p65 antibody (Abcam, Cambridge, MA) at room temperature for 60 min. Negative control was done by using normal mouse-IgG. The precipitated DNA was added 5 M NaCl and heated at 65°C to reverse histone-DNA crosslinks. The immunocomplexes were treated with proteinase K, and DNA fragments were purified for subsequent real-time PCR. ChIP data were normalized to input DNA from each sample. The sequences of specific *nos II* promoter primers were: 5’-AACACGAGGCTGAGCTGAAT-3’ (forward primer) and 5’-TACATGGCATGGGATTTTCC-3’ (reverse primer). The amplified products were confirmed by running 1% agarose gels.

### Enzyme-linked immunosorbent assay (ELISA)

We determined the protein levels of nitrotyrosine with OxiSelect Nitrotyrosine ELISA Kit (Cell Biolabs, San Diego, CA) following the protocol recommended by the manufacturer, and modified for brain tissues. Protein extracted from RVLM was first added to a nitrated BSA preabsorbed EIA plate. After brief incubation, an anti-nitrotyrosine antibody was added, followed by an HRP conjugated secondary antibody. The final absorbance of reaction solution was determined by spectrophotometry using an ELISA microtiter plate reader (Thermo Scientific, Waltham, MA) using 450 nm as the primary wave length. Nitrotyrosine contents were determined by comparing with a standard curve prepared from predetermined nitrated BSA standards.

### Statistical analysis

All values are expressed as mean ± SEM. The averaged value of MSAP or HR calculated every 20 min after administration of test agents or vehicle, the sum total of power density for the LF component in the SAP spectrum over 20 min, and changes in real-time PCR products, protein expression or enzyme activity in RVLM during each phase of experimental brain death, was used for statistical analysis. One-way or two-way ANOVA with repeated measures was used, as appropriate, to assess group means. This was followed by the Scheffé multiple-range test for *post hoc* assessment of individual means. *P <*0.05 was considered to be statistically significant.

## Supporting Information

Figure S1 **Synthesis of NOS II via transcriptional activation of NF-κB in RVLM.** Representative gel depicting NF-κB DNA binding detected by EMSA in nuclear extracts from ventrolateral medulla of rats that received IV administration of LPS (15 mg kg^−1^). In a supershift assay, nuclear extracts were preincubated with an antiserum against NF-κB p50 subunit. A competitive assay with the addition of 100-fold of unlabeled NF-κB oligonucleotide was used to control for non-specific binding. ns denotes non-specific binding.
(TIF)
