# Supplementary figures and images for: A Double-Edged Sword Role for Ubiquitin-Proteasome System in Brain Stem Cardiovascular Regulation During Experimental Brain Death
Source: PLoS One. 2011 Nov 14;6(11):e27404. doi: 10.1371/journal.pone.0027404 (PMC3215722; doi:10.1371/journal.pone.0027404)

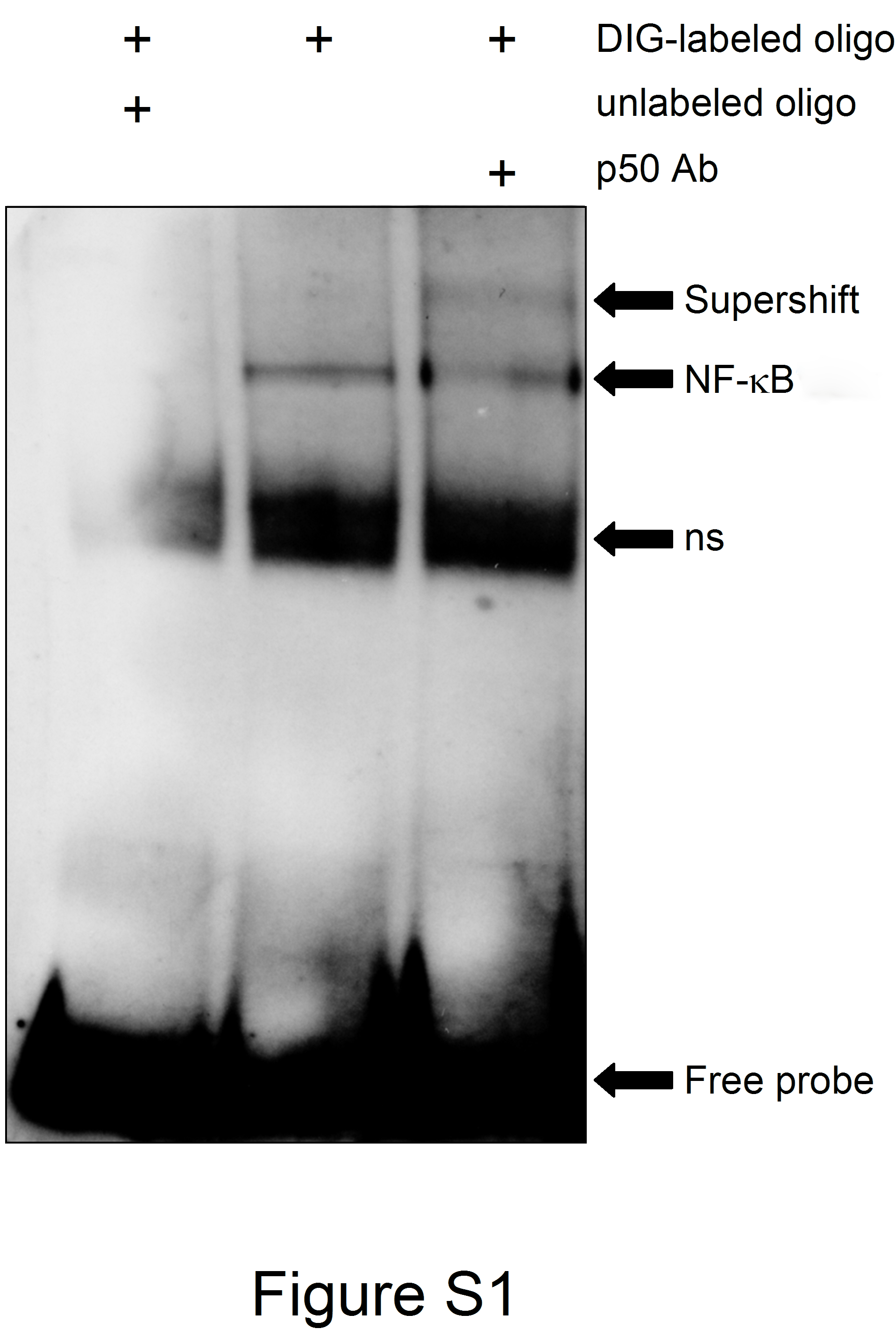

Supplement: Figure S1 — Synthesis of NOS II via transcriptional activation of NF-κB in RVLM. Representative gel depicting NF-κB DNA binding detected by EMSA in nuclear extracts from ventrolateral medulla of rats that received IV administration of LPS (15 mg kg−1). In a supershift assay, nuclear extracts were preincubated with an antiserum against NF-κB p50 subunit. A competitive assay with the addition of 100-fold of unlabeled NF-κB oligonucleotide was used to control for non-specific binding. ns denotes non-specific binding. (TIF) [file pone.0027404.s001.tif]
